# Perception and satisfaction regarding an intradialytic virtual reality exercise program in Brazil

**DOI:** 10.1590/2175-8239-JBN-2024-0133en

**Published:** 2025-01-31

**Authors:** Marcelly Oliveira Pinton, Bruno Valle Pinheiro, Fabrício Sciammarella Barros, Daniele Thomé Silva, Ana Paula de Lima Souza Dias, Maria Clara de Lima Assis, Emanuele Poliana Lawall Gravina, Leda Marília Fonseca Lucinda, Eva Segura Orti, Maycon Moura Reboredo

**Affiliations:** 1 Universidade Federal de Juiz de Fora, Programa de Pós-Graduação em Ciências da Reabilitação e Desempenho Físico, Juiz de Fora, MG, Brazil.; 2 Universidade Federal de Juiz de Fora, Núcleo de Pesquisa em Pneumologia e Terapia Intensiva, Juiz de Fora, MG, Brazil.; 3 Universidade Federal de Juiz de Fora, Faculdade de Medicina, Juiz de Fora, MG, Brazil.; 4 Universidade Federal de Juiz de Fora, Hospital Universitário, Empresa Brasileira de Serviços Hospitalares, Juiz de Fora, MG, Brazil.; 5 Universidad Cardenal Herrera-CEU, CEU Universities, Departamento de Fisioterapia, Valencia, Spain.

**Keywords:** Renal Dialysis, Virtual Reality, Exercise

## Abstract

**Introduction:**

Virtual reality (VR) has been used as an effective tool for improving patient adherence to intradialytic exercise programs. This study evaluated the perception and satisfaction of patients and healthcare professionals regarding an intradialytic VR exercise program.

**Methods:**

The VR protocol included lower limb resistance and endurance training. Patients’ perception and satisfaction, as well as the perception of healthcare professionals, were assessed using questionnaires.

**Results:**

Of the 27 patients who participated in the exercise program, most evaluated the experience as very good and easy to perform. Of the 24 patients who completed the program, most described the experience as beneficial. Most of the healthcare professionals reported that the protocol did not affect their work routine.

**Conclusion:**

The intradialytic VR exercise program was well accepted by patients and healthcare professionals.

## Introduction

Virtual reality (VR) has been studied and described as an effective and safe tool to improve adherence of hemodialysis patients to physical exercise (PE) programs, reducing sedentary behavior^1,2,3,4^. VR, a computer-simulated three-dimensional environment that offers interaction with real-world-like scenarios, is a promising approach in the care of patients with chronic kidney disease, as it diversifies PE practice, rendering it more dynamic and interactive^3,5,6^.

A study conducted in Spain compared the effects of PE using VR with conventional PE in hemodialysis patients. After 16 weeks, patients in both groups had increased muscle strength; however, adherence to the VR program was significantly higher, with no risks. Therefore, in addition to promoting greater adherence, VR represented a strategy for muscle strengthening in these patients^6^.

In another more recent study comparing PE using VR with aerobic training, greater adherence to VR treatment was also observed in hemodialysis patients^2^.

Although VR has proven to be a strategy associated with greater patient adherence to PE programs, offering a more engaging and interactive approach than conventional methods, the perception and satisfaction of hemodialysis patients and healthcare professionals regarding a VR-based PE program have not yet been evaluated in low- and middle-income countries such as Brazil^3,5^. Therefore, this study evaluated patients’ perception and satisfaction regarding an intradialytic VR PE program, as well as the perception of healthcare professionals from the hemodialysis clinic about this program.

## Methods

### Sample

The study was conducted in the hemodialysis sector of the Division of Nephrology at the University Hospital of the *Universidade Federal de Juiz de Fora* (HU-UFJF/EBSERH), between August 2023 and January 2024. It was approved by the HU-UFJF Human Research Ethics Committee (CAAE 67028823.9.0000.5133). All participants who agreed to participate signed an informed consent form.

The sample for this study consisted of adult patients of both sexes undergoing hemodialysis for a minimum of three months, who participated in a randomized clinical trial evaluating the effects of an intradialytic PE program using VR. The following exclusion criteria were considered: vascular access in the lower limbs, presence of cognitive, neurological, musculoskeletal, and osteoarticular disorders that could affect the implementation of the exercise protocol.

Healthcare professionals working in the hemodialysis sector of HU-UFJF/EBSERH were also included.

### Virtual Reality Exercise Protocol

The protocol enabled the performance of lower limb muscle strengthening and endurance exercises directly on the dialysis chair or bed. Patients were positioned so that they could view the computer screen (All in One Inspiron 5430-Dell^©^) placed on a table, allowing the motion sensor (XboX 360 Kinect Sensor- Microsoft Corporation^©^) to capture the movements of their lower limbs (Figures S1 and S2). Each patient used a computer, which allowed for better visualization of the screen (24 inches) and individualized exercise prescription. The movements controlled an avatar in the VR software (*A la caza del tezoro – Universitat Politécnica de València* ^©^), in which the objective was to collect the coins and escape the bombs^2,4,6^. The lower limbs were moved in all directions, including periods of isometry, with the distance between the motion sensor and the patient adjusted to ensure safe ranges of movement. Patients freely alternated the lower limb used to control the avatar. During the first week of training, patients performed two sets of exercises, each lasting three minutes. From the second week onwards, the number of sets increased until they reached six sets of three minutes each. Subsequently, the duration of the sets was extended, reaching a maximum of six sets of six minutes each.

### Experimental Protocol

All patients who started the VR program were subjected to an interview to apply a questionnaire assessing their perception of PE using VR, which included questions about the ease and pleasure of performing the training, the overall quality of the program, and the instructions provided about the training. This questionnaire used a scale ranging from poor (0) to excellent (4). For patients who decided to discontinue the program, the reason for their withdrawal was inquired.

After 12 weeks of training, patients who com­pleted the program underwent a new interview and questionnaire to assess their satisfaction with the VR PE program. The questionnaire included questions about their experience with the training, likelihood of recommending the program, and willingness to continue the protocol. The patients responded to dichotomous questions (yes or no).

Additionally, throughout the 12 weeks of the protocol, the healthcare professionals involved received anonymous questionnaires containing questions about the functioning and feasibility of the VR PE program and its effects on patient health. The healthcare professionals responded to dichotomous questions (yes or no) and multiple-choice questions.

All questionnaires were developed by the researchers themselves, based on previous studies on intradialytic PE protocols^1,4,5,6,7,8^. The final version of the questionnaires was reviewed by three experts in intradialytic exercise (MMR, FSB, and ESO).

### Statistical Analysis

Descriptive statistics were used to evaluate the questionnaires’ results. Data were expressed in absolute numbers and percentages. All analyses were performed using the SPSS 17.0 for Windows program (SPSS Inc. Released, 2008).

## Results

Of the 91 patients undergoing hemodialysis treatment, 49 were not eligible [clinical instability (n = 13); neurological deficit (n = 9); visual impairment (n = 8); amputation (n = 4); lower limb access (n = 3); cognitive impairment (n = 3); others (n = 9)]. Also, 15 patients did not agree to participate (35.7% of those eligible), and 27 were included in the VR PE program. During the 12-week protocol, 3 patients discontinued the training due to preferring traditional endurance training, not finding the experience enjoyable, or fear of hypotension episodes.

Sociodemographic, clinical, and laboratory data are presented in Table S1. The sample consisted mostly of male patients, with a mean age of 60.8 years. The most prevalent etiology of CKD and comorbidity was hypertension.

As shown in Table 1, most patients characterized their perception of PE using VR as very good, except for the quality of the images, which most described as good.

**Table 1:** Evaluation of patients’ perception of intradialytic virtual reality exercise program (n = 27)

Evaluation	Results					
	Image quality	Program interface	Ease of use the program	Enjoyment in using the program	General quality of the program	Instructions provided for the program
Poor	0 (0%)	0 (0%)	1 (3.7%)	2 (7.4%)	0 (0%)	0 (0%)
Acceptable	3 (11.1%)	1 (3.7%)	2 (7.4%)	1 (3.7%)	3 (11.1%)	0 (0%)
Good	10 (37.0%)	9 (33.3%)	9 (33.3%)	4 (14.8%)	5 (18.5%)	10 (37.0%)
Very good	9 (33.3%)	15 (55.6%)	11 (40.7%)	10 (37.0%)	11 (40.7%)	11 (40.7%)
Excellent	5 (18.5%)	2 (7.4%)	4 (14.8%)	10 (37.0%)	8 (29.6%)	6 (22.2%)

**Figure 1:**
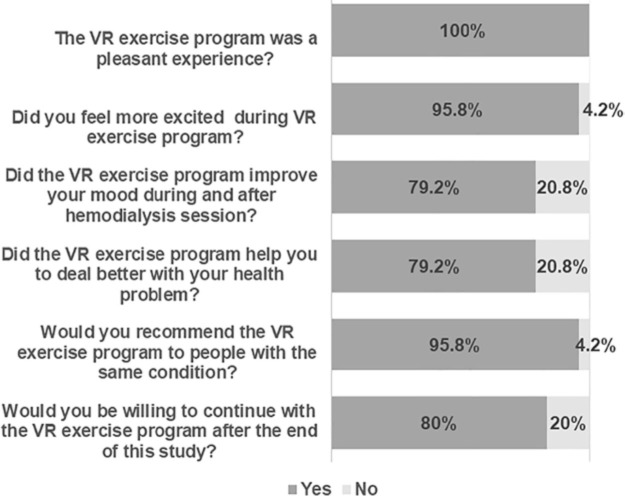
Evaluation of patients’ satisfaction with intradialytic virtual reality (VR) exercise program (n = 24).

The satisfaction assessment of the VR PE program from the 24 patients who completed the protocol is described in Figure 1. The vast majority described the experience as pleasant, observed benefits, recommended it to other patients, and were willing to continue in the program.

Of the 60 professionals working at the hemodialysis clinic, 29 agreed to participate and responded to the questionnaire. The data on the professionals are described in Table S2. The evaluation of the professionals’ perception regarding the functioning and feasibility of the VR PE program is depicted in Table 2. Most professionals reported that the program is viable and had no impact on their routine at the dialysis center. However, the professionals reported some challenges regarding the acceptance of this program by the healthcare team, such as integration into the workflow and changes to the work environment.

**Table 2:** Evaluation of healthcare professionals’ perception regarding the operation and feasibility of implementing an intradialytic virtual reality (VR) exercise program (n = 29)

Questions	Yes n (%)
*Implementation and impact on professional activity* *	
Do you think it is feasible to implement an intradialytic VR exercise program in hemodialysis units?	29 (100)
Do you think the introduction of VR exercise programs in physiotherapy during hemodialysis could negatively impact your responsibilities in the workplace?	4 (13.8)
Do you think the introduction of VR exercise programs in physiotherapy during hemodialysis could negatively impact your daily tasks in the workplace?	3 (10.3)
Do you think the introduction of VR exercise programs in physiotherapy during hemodialysis could negatively affect the organization of the workplace?	7 (24.1)
Do you see opportunities for interdisciplinary collaboration among healthcare teams in implementing this program?	25 (86.2)
*What are the primary advantages you perceive in this approach for patients?* **	
Improvement of quality of life	25 (86.2)
Increase in treatment adherence	14 (48.3)
Improvement in mental health	25 (86.2)
Promotion of physical activity	28 (96.6)
Improvement in cardiovascular function	22 (75.9)
Preservation of muscular function	27 (93.1)
*What challenges do you believe may exist regarding the acceptance of this program by patients?* **	
Level of familiarity with technology	16 (55.2)
Pre-existing health conditions	20 (69.0)
Cultural and linguistic barriers	6 (20.7)
Motivation	15 (51.7)
Physical adaptation	13 (44.8)
*What challenges do you believe may exist regarding the acceptance of this program by the healthcare team?* **	
Workflow integration	17 (58.6)
Change in the work environment	14 (48.3)
Level of knowledge about the effects of the program on patient health	11 (37.9)
Scheduling of activities	1 (3.4)
Lack of environment structure	1 (3.4)
*What additional resources (personnel, equipment, training) do you think would be necessary to successfully implement VR in treatment?* **	
Computers	20 (69.0)
Nurses	5 (17.2)
Physiotherapists	15 (51.7)
Personal protective equipment	5 (17.2)
Training on the program and its effects on patient health for the patients	23 (79.3)
Training on the program and its effects on patient health for the health professionals	18 (62.1)
Furniture suitable for VR-based exercise	1 (3.4)

Notes – *Dichotomous question.

**Multiple-choice question.

## Discussion

In the present study, we observed that patients characterized their perception of PE using VR as very good, described the experience as pleasant, and reported some benefits. In addition, healthcare professionals reported that the program is feasible and has no impact on the routine of the dialysis center.

After the VR PE protocol, we found that most patients felt more energetic and had improved mood. Additionally, patients considered the experience of using VR to be easy and pleasant. Similarly, in the study by Turon´-Skrzypin´ska, it was observed that intradialytic PE using VR led to a reduction in levels of anxiety and depression, as well as reducing the monotony of hemodialysis sessions^7^.

An important finding of this study was that all professionals reported good acceptance for the implementation of VR as a form of PE in hemodialysis centers, with a small percentage stating that VR would have a negative impact on their responsibilities and daily tasks in the work environment. However, the perception of professionals in our study may have been influenced by the presence of regular PE programs at the assessed dialysis center, which have already demonstrated beneficial effects on the quality of life and health of this population. Furthermore, the professionals reported some potential challenges regarding the acceptance of this program by the team, such as integration into the workflow and changes to the work environment. Previous studies have identified barriers to the practice of intradialytic PE, such as resistance from other professionals, lack of funding, human resources and equipment, and concerns about patient safety^8,9,10^.

This study has some limitations, such as the small number of participants and the fact that the protocol was conducted in a single center. Conversely, it evaluated an innovative strategy, which was highly receptive and provided patient satisfaction, in addition to having no impact on the workload of professionals. Clinically, it is important to highlight that PE using VR acts as an adjunct to physiotherapy treatment, requiring the work of a qualified professional for its prescription and monitoring. This monitoring is essential to ensure the safety and effectiveness of the procedure^4,6^.

From the above, it was concluded that a VR PE program during hemodialysis sessions represented a well-accepted strategy among both patients and professionals. In addition, patients characterized their perception of VR as very good, with the majority showing high satisfaction with the program.

## Supplementary Material

The following online material is available for this article:

Table S1 – Clinical, sociodemographic and laboratory data of the study patients.

Table S2 – Data on healthcare professionals from the hemodialysis center.

Figure S1 – Table set up before patient use, showing the equipment (A computer and B: motion sensor).

Figure S2 – Physical exercise program with virtual reality.

## References

[1] Chou HY, Chen SC, Yen TH, Han HM (2020). Effect of a virtual reality-based exercise program on fatigue in
hospitalized Taiwanese end-stage renal disease patients undergoing
hemodialysis.. Clin Nurs Res..

[2] Martínez-Olmos FJ, Gómez-Conesa AA, García-Testal A, Ortega-Pérez-de-Villar L, Valtueña-Gimeno N, Gil-Gómez JA (2022). An intradialytic non-immersive virtual reality exercise
programme: a crossover randomized controlled trial.. Nephrol Dial Transplant..

[3] Qian J, McDonough DJ, Gao Z (2020). The effectiveness of virtual reality exercise on individual’s
physiological, psychological and rehabilitative outcomes: a systematic
review.. Int J Environ Res Public Health..

[4] García-Testal A, Martínez-Olmos FJ, Gil-Gómez JA, López-Tercero V, Lahoz-Cano L, Hervás-Marín D (2022). Healthcare.

[5] Omonaiye O, Smyth W, Nagle C (2021). Impact of virtual reality interventions on haemodialysis
patients: a scoping review.. J Ren Care..

[6] Segura-Ortí E, García-Testal A (2019). Intradialytic virtual reality exercise: increasing physical
activity through technology.. Semin Dial..

[7] Turon´-Skrzypin´ska A, Tomska N, Mosiejczuk H, Rył A, Szylin´ska A, Marchelek-Mys´liwiec M (2023). Impact of virtual reality exercises on anxiety and depression in
hemodialysis patients.. Sci Rep..

[8] Barros FS, Pinheiro BV, Lucinda LMF, Rezende GF, Segura-Ortí E, Reboredo MM (2021). Exercise training during hemodialysis in Brazil: a national
survey.. Artif Organs..

[9] Ma S, Lui J, Brooks D, Parsons TL (2012). The availability of exercise rehabilitation programs in
hemodialysis centres in Ontario.. CANNT J..

[10] Kutner NG (2010). Rehabilitation in the renal population: barriers to
access.. Semin Nephrol..

