# Supplementary material for: Perception and satisfaction regarding an intradialytic virtual reality exercise program in Brazil
Source: J Bras Nefrol. 2025 Jan 31;47(2):e20240133. doi: 10.1590/2175-8239-JBN-2024-0133en (PMC11831697; doi:10.1590/2175-8239-JBN-2024-0133en)
Supplement: Supplementary file 1 [file 2175-8239-jbn-47-2-e20240133-suppl3.pdf]

Braz. J. Nephrol. ~~*(J. Bras. Nefrol.)*~~
https://doi.org/10.1590/2175-8239-JBN-2024-0133en

Supplementary Material to "Perception and satisfaction regarding an intradialytic virtual reality exercise program in Brazil"

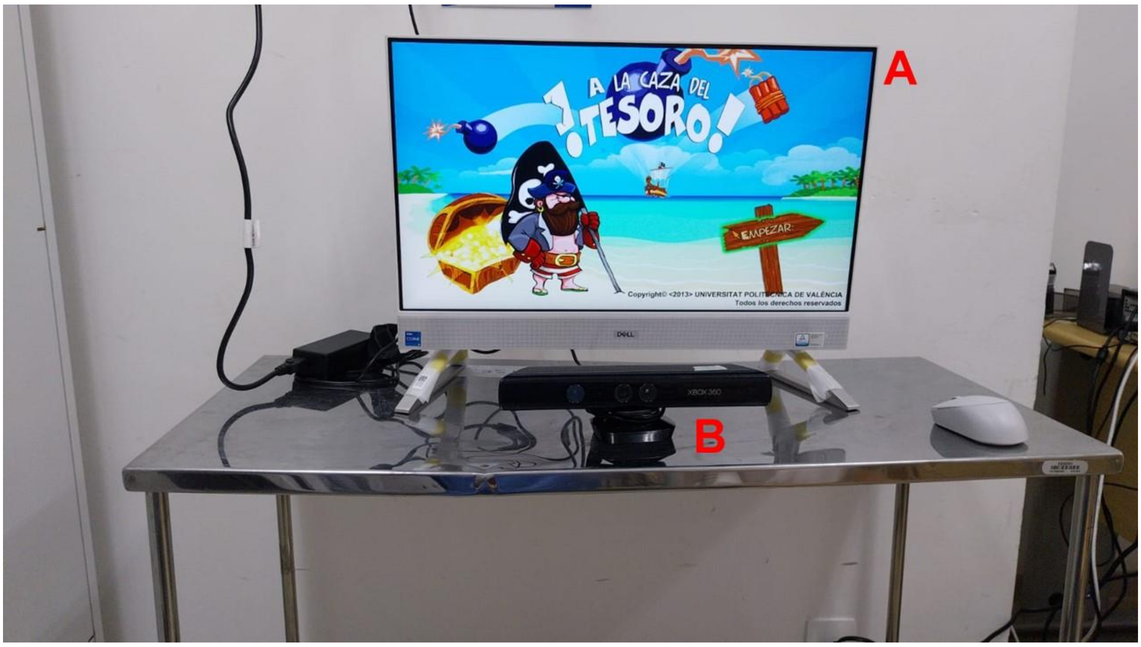

Figure S1. Table set up before patient use, showing the equipment (A - computer, B – motion tracking system).
