# Supplementary material for: Perception and satisfaction regarding an intradialytic virtual reality exercise program in Brazil
Source: J Bras Nefrol. 2025 Jan 31;47(2):e20240133. doi: 10.1590/2175-8239-JBN-2024-0133en (PMC11831697; doi:10.1590/2175-8239-JBN-2024-0133en)
Supplement: Supplementary file 3 [file 2175-8239-jbn-47-2-e20240133-suppl1.pdf]

Braz. J. Nephrol.
https://doi.org/10.1590/2175-8239-JBN-2024-0133en

Supplementary Material to "Perception and satisfaction regarding an intradialytic virtual reality exercise program in Brazil"

TABLE S1 Clinical, sociodemographic, and laboratory data of the participants.

**Variables**	**n = 27**
Sociodemographic data	
Age (years)*	60.8 ± 12.4
Male, n (%)	15 (55.6)
Educational level (years)*	8.8 ± 3.9
Monthly family income (R$)**	2600 (1320 - 2600)
Time on dialysis (months)**	66 (45 – 116)
Comorbidities, n (%)	
Hypertension	23 (85.2)
Obesity	15 (55.6)
Diabetes mellitus	7 (25.9)
Cardiovascular disease	6 (22.2)
Retinopathy	3 (11.1)
Hyperparathyroidism	3 (11.1)
Hypothyroidism	3 (11.1)
Chronic kidney disease etiology, n (%)	
Hypertension	7 (25.9)
Diabetes mellitus	5 (18.5)
Glomerulopathies	5 (18.5)
Others	4 (14.8)
Unknown	6 (22.2)
Laboratory data	
Hemoglobin (mg/dL)**	10.6 (9.6 – 10.8)
Creatinine (mg/dL)*	11.1 ± 2.5
Albumin (g/dL)*	4.9 ± 0.7

Hemodialysis efficiency index*	1.6 ± 0.2
Body mass index (Kg/m²)*	27.2 ± 5.8

*Data are expressed as the mean ± standard deviation.

**Data are expressed as the median (interquartile range).
