# Supplementary material for: Perception and satisfaction regarding an intradialytic virtual reality exercise program in Brazil
Source: J Bras Nefrol. 2025 Jan 31;47(2):e20240133. doi: 10.1590/2175-8239-JBN-2024-0133en (PMC11831697; doi:10.1590/2175-8239-JBN-2024-0133en)
Supplement: Supplementary file 4 [file 2175-8239-jbn-47-2-e20240133-suppl2.pdf]

Braz. J. Nephrol
https://doi.org/10.1590/2175-8239-JBN-2024-0133en

Supplementary Material to "Perception and satisfaction regarding an intradialytic virtual reality exercise program in Brazil"

TABLE S2 Healthcare professionals' data at the hemodialysis unit.

**Variables**	**n = 29**
Professionals, n (%)	
Nursing technician	14 (48.3)
Nurse	8 (27.6)
Physiotherapist	2 (6.9)
Social worker	2 (6.9)
Nutritionist	1 (3.4)
Dentist	1 (3.4)
Psychologist	1 (3.4)
Demographic and experience in the field data	
Age (years)*	41.1 ± 5.1
Length of time working in the hemodialysis field (years)*	7.7 ± 6.7
Length of time working at the hemodialysis center (years)*	5.8 ± 4.6

*Data are expressed as the mean ± standard deviation.
