# Supplementary material for: Perception and satisfaction regarding an intradialytic virtual reality exercise program in Brazil
Source: J Bras Nefrol. 2025 Jan 31;47(2):e20240133. doi: 10.1590/2175-8239-JBN-2024-0133en (PMC11831697; doi:10.1590/2175-8239-JBN-2024-0133en)
Supplement: Supplementary file 5 [file 2175-8239-jbn-47-2-e20240133-suppl7.pdf]

Braz. J. Nephrol.
https://doi.org/10.1590/2175-8239-JBN-2024-0133pt

Material Suplementar para "Percepção e satisfação sobre um programa de exercício físico intradialítico utilizando realidade virtual no Brasil"

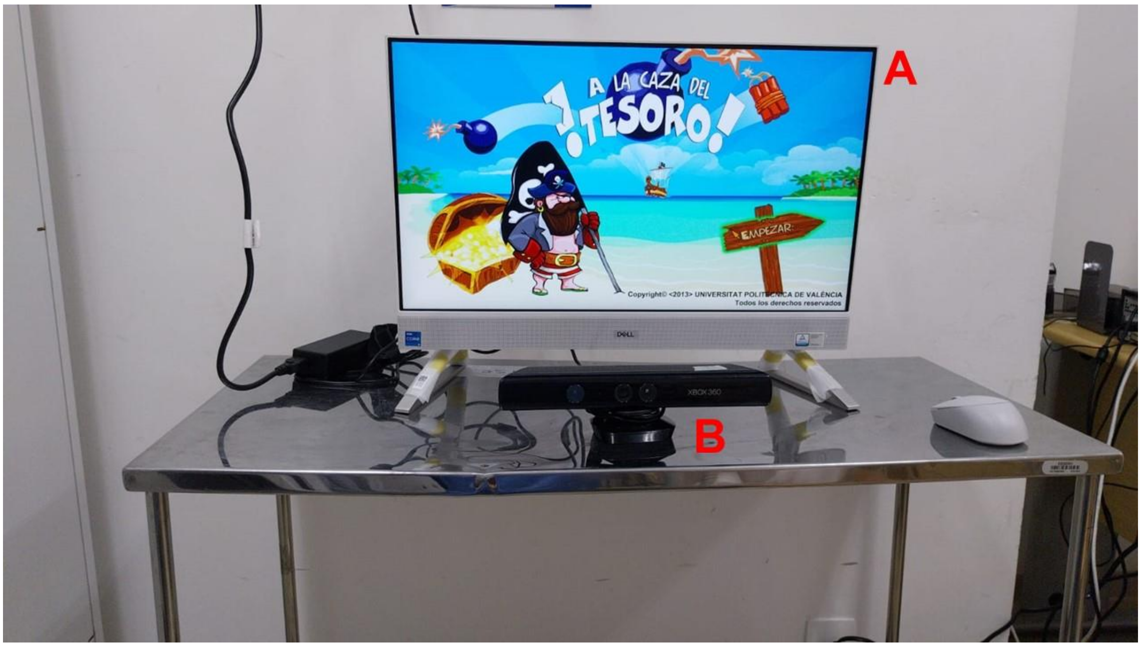

Figura S1. Mesa montada antes do uso pelo paciente, mostrando os equipamentos (A: computador; B: sensor de movimentos).
