# Supplementary material for: Perception and satisfaction regarding an intradialytic virtual reality exercise program in Brazil
Source: J Bras Nefrol. 2025 Jan 31;47(2):e20240133. doi: 10.1590/2175-8239-JBN-2024-0133en (PMC11831697; doi:10.1590/2175-8239-JBN-2024-0133en)
Supplement: Supplementary file 7 [file 2175-8239-jbn-47-2-e20240133-suppl5.pdf]

Braz. J. Nephrol.
https://doi.org/10.1590/2175-8239-JBN-2024-0133pt

Material Suplementar para "Percepção e satisfação sobre um programa de exercício físico intradialítico utilizando realidade virtual no Brasil"

TABELA S1 Dados clínicos, sociodemográficos e laboratoriais dos pacientes participantes do estudo.

**Variáveis**	**n = 27**
Dados sociodemográficos	
Idade (anos)*	60,8 ± 12,4
Sexo masculino, n (%)	15 (55,6)
Escolaridade (anos)*	8,8 ± 3,9
Renda familiar mensal (R$)**	2600 (1320-2600)
Tempo em diálise (meses)**	66 (45-116)
Comorbidades, n (%)	
Hipertensão arterial	23 (85,2)
Obesidade	15 (55,6)
Diabetes *mellitus*	7 (25,9)
Doença cardiovascular	6 (22,2)
Retinopatia	3 (11,1)
Hiperparatireoidismo	3 (11,1)
Hipotireoidismo	3 (11,1)
Etiologia da doença renal crônica, n (%)	
Hipertensão arterial	7 (25,9)
Diabetes *mellitus*	5 (18,5)
Glomerulopatias	5 (18,5)
Outros	4 (14,8)
Indeterminada	6 (22,2)
Dados laboratoriais	
Hemoglobina (mg/dL)**	10,6 (9,6-10,8)

Creatinina (mg/dL)*	11,1 ± 2,5
Albumina (g/dL)*	4,9 ± 0,7
Índice de eficácia de diálise*	1,6 ± 0,2
Índice de massa corporal (kg/m²)*	27,2 ± 5,8

* Dados expressos em média ± desvio-padrão. ** Dados expressos em mediana (intervalo interquartil).
