# Supplementary material for: Perception and satisfaction regarding an intradialytic virtual reality exercise program in Brazil
Source: J Bras Nefrol. 2025 Jan 31;47(2):e20240133. doi: 10.1590/2175-8239-JBN-2024-0133en (PMC11831697; doi:10.1590/2175-8239-JBN-2024-0133en)
Supplement: Supplementary file 8 [file 2175-8239-jbn-47-2-e20240133-suppl6.pdf]

Braz. J. Nephrol.

https://doi.org/10.1590/2175-8239-JBN-2024-0133pt

Material Suplementar para "Percepção e satisfação sobre um programa de exercício físico intradialítico utilizando realidade virtual no Brasil"

Tabela S2 Dados dos profissionais de saúde do centro de hemodiálise.

**Variáveis**	**n = 29**
Profissionais, n (%)	
Técnico de enfermagem	14 (48,3)
Enfermeiro	8 (27,6)
Fisioterapeuta	2 (6,9)
Assistente social	2 (6,9)
Nutricionista	1 (3,4)
Cirurgião-dentista	1 (3,4)
Psicólogo	1 (3,4)
Dados demográficos e de experiência na área	
Idade (anos)*	41,1 ± 5,1
Tempo de trabalho na área de hemodiálise (anos)*	7,7 ± 6,7
Tempo de trabalho nesse centro de diálise (anos)*	5,8 ± 4,6

* Dados expressos em média ± desvio-padrão.
