# Supplementary material for: Perception and satisfaction regarding an intradialytic virtual reality exercise program in Brazil
Source: J Bras Nefrol. 2025 Jan 31;47(2):e20240133. doi: 10.1590/2175-8239-JBN-2024-0133en (PMC11831697; doi:10.1590/2175-8239-JBN-2024-0133en)
Supplement: Translation

# Translation: Percepção e satisfação sobre um programa de exercício físico intradialítico utilizando realidade virtual no Brasil

**DOI:** 10.1590/2175-8239-JBN-2024-0133pt

**Authors:** Marcelly Oliveira Pinton, Bruno Valle Pinheiro, Fabrício Sciammarella Barros, Daniele Thomé Silva, Ana Paula de Lima Souza Dias, Maria Clara de Lima Assis, Emanuele Poliana Lawall Gravina, Leda Marília Fonseca Lucinda, Eva Segura Orti, Maycon Moura Reboredo

**Affiliations:** 1 Universidade Federal de Juiz de Fora, Programa de Pós-Graduação em Ciências da Reabilitação e Desempenho Físico, Juiz de Fora, MG, Brasil.; 2 Universidade Federal de Juiz de Fora, Núcleo de Pesquisa em Pneumologia e Terapia Intensiva, Juiz de Fora, MG, Brasil.; 3 Universidade Federal de Juiz de Fora, Faculdade de Medicina, Juiz de Fora, MG, Brasil.; 4 Universidade Federal de Juiz de Fora, Hospital Universitário, Empresa Brasileira de Serviços Hospitalares, Juiz de Fora, MG, Brasil.; 5 Universidad Cardenal Herrera-CEU, CEU Universities, Departamento de Fisioterapia, Valência, Espanha.

**Keywords:** Diálise Renal, Realidade Virtual, Exercício Físico

## Abstract

**Introdução:**

A realidade virtual (RV) tem se mostrado uma ferramenta eficaz para melhorar a adesão de pacientes a programas de exercícios intradialíticos. Este estudo avaliou a percepção e satisfação dos pacientes e profissionais de saúde sobre um programa de exercício físico intradialítico utilizando a RV.

**Método:**

O protocolo com RV incluiu exercícios de fortalecimento e resistência dos membros inferiores. A percepção e satisfação dos pacientes, além da percepção dos profissionais de saúde, foram avaliadas por questionários.

**Resultados:**

Dos 27 pacientes que participaram do programa de exercícios, a maioria avaliou a experiência como muito boa e de fácil execução. Dos 24 pacientes que completaram o programa, a maioria descreveu a experiência como benéfica. A maioria dos 29 profissionais avaliados relatou que o protocolo não afetou a rotina de trabalho.

**ConclusÃO:**

O exercício intradialítico com RV foi bem aceito pelos pacientes e profissionais de saúde.

## Introdução

A realidade virtual (RV) tem sido estudada e descrita como uma ferramenta eficaz e segura para melhorar a adesão dos pacientes em hemodiálise a programas de exercício físico (EF), reduzindo o comportamento sedentário^1,2,3,4^. A RV, um ambiente tridimensional simulado por computador que oferece interação com cenários semelhantes ao mundo real, é uma abordagem promissora no atendimento de pacientes com doença renal crônica, pois diversifica a prática de EF, tornando-o mais dinâmico e interativo^3,5,6^.

Um estudo conduzido na Espanha comparou os efeitos do EF utilizando RV com EF convencional em pacientes em hemodiálise. Após 16 semanas, em ambos os grupos os pacientes tiveram aumento da força muscular, mas a adesão ao programa de RV foi significativamente maior, não apresentando riscos. Portanto, além de promover maior adesão, a RV representou uma estratégia para o fortalecimento muscular desses pacientes^6^.

Em outro estudo mais recente, que comparou o EF utilizando a RV com o treinamento aeróbico, também foi observada maior adesão ao tratamento com RV em pacientes em hemodiálise^2^.

Embora a RV tenha se mostrado uma estratégia associada a maior adesão dos pacientes a programas de EF, oferecendo uma abordagem mais atrativa e interativa do que os métodos convencionais, ainda não foi avaliada a percepção e satisfação dos pacientes em hemodiálise e profissionais de saúde sobre um programa de EF utilizando a RV em países de baixa e média renda, como o Brasil^3,5^. Portanto, este estudo avaliou a percepção e satisfação dos pacientes sobre um programa de EF intradialítico utilizando a RV, bem como a percepção dos profissionais da saúde da clínica de hemodiálise sobre o programa.

## Métodos

### Amostra

O estudo foi conduzido no setor de hemodiálise da Unidade do Sistema Urinário do Hospital Universitário da Universidade Federal de Juiz de Fora (HU-UFJF/EBSERH), no período de agosto de 2023 a janeiro de 2024, e foi aprovado pelo Comitê de Ética em pesquisa com seres humanos do HU-UFJF (CAAE 67028823.9.0000.5133). Todos os participantes que concordaram em participar assinaram o termo de consentimento livre e esclarecido.

A amostra deste estudo foi composta por pacientes adultos, de ambos os sexos, em hemodiálise por um período mínimo de três meses, participantes de um ensaio clínico randomizado que avaliou os efeitos de um programa de EF intradialítico utilizando RV. Foram considerados os seguintes critérios de exclusão: acesso vascular nos membros inferiores, presença de distúrbios cognitivos, neurológicos, musculoesqueléticos e osteoarticulares que pudessem afetar a aplicação do protocolo de exercício.

Foram incluídos também os profissionais da área da saúde que atuam no setor de hemodiálise do HU-UFJF/EBSERH.

### Protocolo de Exercício com Realidade Virtual

O protocolo permitiu a realização de exercícios de fortalecimento e resistência dos músculos dos membros inferiores na própria cadeira ou cama de diálise, com o paciente posicionado de forma que conseguisse visualizar a tela do computador (*All in One Inspiron 5430-Dell* ^©^) apoiado em uma mesa, e permitisse que o sensor de movimento (*XboX 360 Kinect Sensor- Microsoft Corporation* ^©^) captasse os movimentos dos membros inferiores (Figuras S1 e S2). Cada paciente utilizou um computador, o que permitiu melhor visualização da tela (24 polegadas) e uma prescrição individualizada do exercício. Os movimentos controlavam um avatar do software de RV (*A la caza del tezoro – Universidat Politécnica de València* ^©^), no qual o objetivo era capturar as moedas e fugir das bombas^2,4,6^. Os membros inferiores foram movimentados para todas as direções, englobando períodos de isometria, sendo que a distância entre o sensor de movimento e o paciente foi ajustada para garantir amplitudes de movimento seguras. Os pacientes alternaram livremente o membro inferior utilizado para controlar o avatar. Durante a primeira semana de treinamento, os pacientes realizaram duas séries de exercícios de três minutos cada, e a partir da segunda semana, ocorreram incrementos no número de séries até atingir seis séries de três minutos cada. Na sequência, a duração das séries foi aumentada, chegando a, no máximo, seis séries de seis minutos cada.

### Protocolo Experimental

Todos os pacientes que iniciaram o programa de RV foram submetidos a uma entrevista para aplicação de um questionário de avaliação da percepção sobre o EF utilizando a RV, que incluiu questões sobre a facilidade e o prazer em realizar o treinamento, qualidade geral do programa e instruções dadas sobre o treinamento. Nesse questionário, foi utilizada uma escala que variou de ruim (0) a excelente (4). Aos pacientes que desistiram de participar do programa, foi questionado o motivo da desistência.

Após 12 semanas de treinamento, os pacientes que completaram o programa foram submetidos a uma nova entrevista e aplicação de questionário para avaliar a satisfação sobre o programa de EF utilizando a RV, que incluiu questões sobre a experiência com o treinamento, probabilidade de recomendar o programa e disposição em continuar o protocolo. Os pacientes responderam a questões dicotômicas (sim ou não).

Além disso, ao longo das 12 semanas do protocolo, os profissionais de saúde envolvidos receberam questionários anônimos contendo perguntas sobre o funcionamento e a viabilidade do programa de EF utilizando a RV e seus efeitos para a saúde do paciente. Os profissionais de saúde responderam a questões dicotômicas (sim ou não) e questões de múltipla escolha.

Todos os questionários foram elaborados pelos próprios pesquisadores, com base em estudos prévios sobre protocolos de EF intradialíticos^1,4,5,6,7,8^. A versão final dos questionários foi revisada por três especialistas em exercícios intradialíticos (MMR, FSB e ESO).

### Análise Estatística

Foi utilizada estatística descritiva para avaliar os resultados dos questionários. Os dados foram expressos em números absolutos e porcentagens. Todas as análises foram realizadas no programa SPSS 17.0 for Windows (SPSS Inc. Released, 2008).

## Resultados

Dos 91 pacientes que estavam em tratamento hemodialítico, 49 pacientes não foram elegíveis [instabilidade clínica (n = 13); déficit neurológico (n = 9); déficit visual (n = 8); amputação (n = 4); acesso em membros inferiores (n = 3); déficit cognitivo (n = 3); outros (n = 9)], 15 não concordaram em participar (35,7% dos elegíveis) e 27 foram incluídos no programa de EF utilizando a RV. Durante o protocolo de 12 semanas, 3 pacientes interromperam o treinamento, porque preferiram o treinamento resistido tradicional, não acharam a experiência agradável ou por medo de ocorrer episódios de hipotensão.

Os dados sociodemográficos, clínicos e laboratoriais estão demonstrados na Tabela S1. A amostra foi composta, na maioria, por pacientes do sexo masculino, com média de idade de 60,8 anos. A etiologia da DRC e a comorbidade mais prevalentes foi a hipertensão arterial.

Como pode ser observado na Tabela 1, a maioria dos pacientes caracterizou a percepção sobre o EF utilizando a RV como muito boa, exceto para a qualidade das imagens, que a maioria descreveu como boa.

**Tabela 1:** Avaliação da percepção dos pacientes sobre o exercício físico intradialítico utilizando a realidade virtual (n = 27)

Avaliação	Resultados					
	Qualidade das imagens	Interface do programa	Facilidade em utilizar o programa	Prazer em usar o programa	Qualidade geral do programa	Instruções dadas sobre o programa
Ruim	0 (0%)	0 (0%)	1 (3,7%)	2 (7,4%)	0 (0%)	0 (0%)
Razoável	3 (11,1%)	1 (3,7%)	2 (7,4%)	1 (3,7%)	3 (11,1%)	0 (0%)
Bom	10 (37,0%)	9 (33,3%)	9 (33,3%)	4 (14,8%)	5 (18,5%)	10 (37,0%)
Muito bom	9 (33,3%)	15 (55,6%)	11 (40,7%)	10 (37,0%)	11 (40,7%)	11 (40,7%)
Excelente	5 (18,5%)	2 (7,4%)	4 (14,8%)	10 (37,0%)	8 (29,6%)	6 (22,2%)

A avaliação da satisfação sobre o programa de EF utilizando a RV dos 24 pacientes que completaram o protocolo está descrita na Figura 1. A grande maioria descreveu a experiência como agradável, observou benefícios, recomendou para outros pacientes e estava disposta a continuar no programa.

**Figura 1:** Avaliação da satisfação dos pacientes sobre o programa de exercício físico intradialítico utilizando realidade virtual (RV) (n = 24).

Dos 60 profissionais que atuavam na clínica de hemodiálise, 29 concordaram em participar e responderam ao questionário. Os dados dos profissionais estão descritos na Tabela S2. A avaliação da percepção dos profissionais sobre o funcionamento e a viabilidade do programa de EF utilizando a RV está descrita na Tabela 2. A maioria dos profissionais relatou que o programa é viável e que não impactou sua rotina no centro de diálise. Entretanto, os profissionais relataram alguns desafios quanto à aceitação desse programa por parte da equipe de saúde, como integração ao fluxo de trabalho e alteração do ambiente de trabalho.

**Tabela 2:** Avaliação da percepção dos profissionais sobre o funcionamento e a viabilidade da implementação de um programa de exercício físico intradialítico utilizando a realidade virtual (RV) (n = 29)

Questões	Simn (%)
*Implementação e impacto na atividade profissional* *
Acha viável a implementação de um programa de exercício físico intradialítico utilizando a RV em clínicas de hemodiálise?	29 (100)
Acha que a introdução de programas de exercício utilizando a RV no tratamento fisioterapêutico durante a hemodiálise poderia afetar suas responsabilidades no ambiente de trabalho de forma negativa?	4 (13,8)
Acha que a introdução de programas de exercício utilizando a RV no tratamento fisioterapêutico durante a hemodiálise poderia afetar suas tarefas diárias no ambiente de trabalho de forma negativa?	3 (10,3)
Acha que a introdução de programas de exercício utilizando a RV no tratamento fisioterapêutico durante a hemodiálise poderia afetar a organização do ambiente de trabalho de forma negativa?	7 (24,1)
Você vê oportunidades de colaboração interdisciplinar entre a equipe de saúde na implementação desse programa?	25 (86,2)
*Quais os principais benefícios que você enxerga nessa abordagem para os pacientes?* **	
Melhora da qualidade de vida	25 (86,2)
Aumento da adesão ao tratamento	14 (48,3)
Melhora da saúde mental	25 (86,2)
Estímulo à atividade física	28 (96,6)
Melhora da função cardiovascular	22 (75,9)
Manutenção da função muscular	27 (93,1)
*Quais desafios você acredita que possam existir quanto à aceitação desse programa por parte dos pacientes?* **	
Nível de familiaridade com a tecnologia	16 (55,2)
Condições de saúde pré-existentes	20 (69,0)
Barreiras culturais e linguísticas	6 (20,7)
Motivação	15 (51,7)
Adaptação física	13 (44,8)
*Quais desafios você acredita que possam existir quanto à aceitação desse programa por parte da equipe de saúde?* **	
Integração no fluxo de trabalho	17 (58,6)
Alteração do ambiente de trabalho	14 (48,3)
Nível de conhecimento sobre os efeitos do programa na saúde do paciente	11 (37,9)
Horário das atividades	1 (3,4)
Falta de estrutura do ambiente	1 (3,4)
Quais recursos adicionais (pessoal, equipamentos, treinamento) você acha que seriam necessários para implementar com sucesso a RV no tratamento?**	
Computadores	20 (69,0)
Enfermeiros	5 (17,2)
Fisioterapeutas	15 (51,7)
Equipamentos de proteção individual	5 (17,2)
Treinamento para os pacientes sobre o programa e os efeitos dele na saúde do paciente	23 (79,3)
Treinamento para os profissionais de saúde sobre o programa e os efeitos dele na saúde do paciente	18 (62,1)
Mobiliário mais adequado	1 (3,4)

Notas – *Questão dicotômica.

**Questão de múltipla escolha.

## Discussão

No presente estudo, observamos que os pacientes caracterizaram a percepção sobre o EF utilizando a RV como muito boa, descreveram a experiência como agradável e relataram alguns benefícios. Além disso, os profissionais de saúde relataram que o programa é viável e sem impacto na rotina do centro de diálise.

Após o protocolo de EF utilizando a RV, detectamos que a maioria dos pacientes se sentiu mais disposta e teve melhora no humor. Adicionalmente, os pacientes consideraram a experiência em utilizar a RV como fácil e agradável. Da mesma forma, no estudo de Turon´-Skrzypin´ska foi observado que o EF intradialítico com a RV promoveu redução dos níveis de ansiedade e depressão, além de diminuir a monotonia das sessões de hemodiálise^7^.

Um importante achado deste estudo foi que todos os profissionais relataram boa aceitação para a implementação da RV como forma de EF nos centros de hemodiálise, sendo que um pequeno percentual relatou que a RV teria impacto negativo em suas responsabilidades e tarefas diárias no ambiente de trabalho. Entretanto, a percepção dos profissionais no nosso estudo pode ter sido influenciada pela presença de programas de EF usuais no centro de diálise avaliado, que já demonstraram efeitos benéficos na qualidade de vida e saúde dessa população. Além disso, os profissionais relataram alguns possíveis desafios quanto à aceitação desse programa por parte da equipe, como integração ao fluxo de trabalho e alteração do ambiente de trabalho. Estudos anteriores identificaram barreiras para a prática de EF intradialítico, como a resistência de outros profissionais, falta de financiamento, recursos humanos e equipamentos, e preocupações com a segurança do paciente^8,9,10^.

Este estudo apresenta algumas limitações, como o número reduzido de participantes e a realização do protocolo em um único centro. Por outro lado, avaliou uma estratégia inovadora, que teve alta receptividade e proporcionou satisfação aos pacientes, além de não representar impacto na carga de trabalho dos profissionais. Clinicamente, é relevante destacar que o EF com a RV atua como um coadjuvante no tratamento fisioterapêutico, requerendo a atuação de um profissional capacitado para sua prescrição e monitoramento. Esse acompanhamento é essencial para garantir a segurança e a eficácia do procedimento^4,6^.

Pelo exposto, concluímos que um programa de EF com RV durante as sessões de hemodiálise representou uma estratégia bem aceita pelos pacientes e profissionais. Além disso, os pacientes caracterizaram a percepção sobre a RV como muito boa e a maioria demonstrou alta satisfação com o programa.

## Material Suplementar

O seguinte material online está disponível para o presente artigo:

Tabela S1 – Dados clínicos, sociodemográficos e laboratoriais dos pacientes participantes do estudo.

Tabela S2 – Dados dos profissionais de saúde do centro de hemodiálise.

Figura S1 – Mesa montada antes do uso pelo paciente, mostrando os equipamentos (A computadore B: sensor de movimentos).

Figura S2 – Programa de exercício físico com realidade virtual.
